# Update: Temporary Total Depletion of U.S. Licensed Yellow Fever Vaccine for Civilian Travelers Addressed by Investigational New Drug Use of Imported Stamaril Vaccine

**DOI:** 10.15585/mmwr.mm6629a4

**Published:** 2017-07-28

**Authors:** Mark D. Gershman, Mark J. Sotir

**Affiliations:** 1 Division of Global Migration and Quarantine, National Center for Emerging and Zoonotic Infectious Diseases, CDC.

Sanofi Pasteur, the manufacturer of the only yellow fever vaccine (YF-VAX) licensed in the United States, has announced that their stock of YF-VAX is totally depleted as of July 24, 2017. YF-VAX for civilian use will be unavailable for ordering from Sanofi Pasteur until mid-2018, when their new manufacturing facility is expected to be completed. However, YF-VAX might be available at some clinics for several months, until remaining supplies at those sites are exhausted. In anticipation of this temporary total depletion, in 2016, Sanofi Pasteur submitted an expanded access investigational new drug application to the Food and Drug Administration to allow for importation and use of Stamaril. The Food and Drug Administration accepted Sanofi Pasteur’s application in October 2016.

Manufactured by Sanofi Pasteur in France, Stamaril is not licensed in the United States, but is licensed and distributed in approximately 70 countries, and has comparable efficacy and safety to YF-VAX (*1*). During the interim period until YF-VAX is available again for use in the United States, Stamaril will be available in a limited number of designated clinics, selected to provide access to vaccine in U.S. states and certain territories (*1*). Clinicians and travelers can find clinics offering Stamaril vaccine and those clinics that might have remaining doses of YF-VAX online at https://wwwnc.cdc.gov/travel/yellow-fever-vaccination-clinics/search. Consideration will be given to adding more clinics if critical gaps in vaccine access are identified. CDC and Sanofi Pasteur continue to collaborate on contingency planning to address this situation.

Information about which countries require yellow fever vaccination for entry and for which countries CDC recommends yellow fever vaccination is available at https://wwwnc.cdc.gov/travel/. Updates regarding yellow fever vaccine will be available on CDC’s Travelers’ Health website (https://wwwnc.cdc.gov/travel/) as well as Sanofi Pasteur’s website (http://www.sanofipasteur.us/vaccines/yellowfevervaccine).
